# A novel assay for quantifying Gn–LRP1–mediated neutralizing antibodies against severe fever with thrombocytopenia syndrome virus

**DOI:** 10.1080/22221751.2026.2665001

**Published:** 2026-04-24

**Authors:** Li-Fen Hu, Meng-Ya Tu, Ding-Ye Zhang, Meng-Rui Sun, Si-Yang Liu, Yu-Yao Li, Meng-Yu Liu, Yidan Wang, Beng-Lee Lim, Jia-Bin Li, Chee Wah Tan, Lin-Fa Wang

**Affiliations:** a Department of Infectious Diseases, the First Affiliated Hospital of Anhui Medical University, Hefei, People’s Republic of China; b Anhui Province Key Laboratory of Infectious Diseases, Anhui Medical University, Hefei, People’s Republic of China; c Department of Infectious Diseases, Public Health Clinical Center of Anhui Province, Hefei, People’s Republic of China; d Programme in Emerging Infectious Diseases, Duke-National University of Singapore Medical School, Singapore, Singapore; e Shanghai Frontiers Science Center of Genome Editing and Cell Therapy, Synthetic Biology and Biomedical Engineering Laboratory, Biomedical Synthetic Biology Research Center, Shanghai Key Laboratory of Regulatory Biology, Institute of Biomedical Sciences and School of Life Sciences, East China Normal University, Shanghai, People’s Republic of China; f Infectious Diseases Translational Research Programme, Department of Microbiology and Immunology, Yong Loo Lin School of Medicine, National University of Singapore, Singapore, Singapore

**Keywords:** Severe fever with thrombocytopenia syndrome, neutralizing antibody, virus neutralization test, conventional virus neutralization test, low-density lipoprotein receptor-related protein 1

## Abstract

Accurate and efficient determination of neutralizing antibody (nAb) is critical for assessing individual immune status of severe fever with thrombocytopenia syndrome (SFTS) and identifying key ecological reservoirs of the SFTS virus (SFTSV). However, conventional virus neutralization tests (cVNT) require live virus and are limited by their inability to support rapid, high-throughput screening. Here, we report a novel SFTSV virus neutralization test targeting the interaction between the SFTSV Gn protein and its receptor (LRP1). The test, which has been validated with SFTS patient plasma, achieves a sensitivity of 96.79% and a specificity of 100%. It also shows good correlation with cVNT, with an *R²* value of 0.8902 in head-to-head comparison. This platform offers a safe, rapid, and high-throughput alternative for large-scale population screening and the real-time monitoring of immune responses in SFTS patients.

## Introduction

Severe fever with thrombocytopenia syndrome (SFTS) is a significant emerging infectious disease caused by the SFTS virus (SFTSV), which belongs to the genus *Bandavirus* in the family *Phenuiviridae* [1, 2]. First identified in China in 2009 during the investigation of an outbreak with high morbidity, SFTS has now emerged as a major public health threat in East Asia, affecting countries like China, South Korea, and Japan. Sporadic cases have also been reported in other Asian countries, including Vietnam, Myanmar, and Thailand [3-5]. The risk and impact of SFTSV are increasing, transitioning from an endemic tick-borne disease to a viral haemorrhagic fever capable of causing larger-scale outbreaks due to its multiple potential transmission routes [6]. Beyond tick-borne transmission, SFTSV also demonstrates substantial potential for human-to-human transmission through direct contact with infected blood or bodily fluids, complicating outbreak control and increasing its public health risk [6, 7].

Neutralizing antibody (nAb) detection is essential for assessing individual immune status, estimating asymptomatic infection rates, and tracing transmission chains. However, conventional virus neutralization test (cVNT) for nAbs requires live virus and cell culture in high-level biosafety facilities, and are often less quantitative, less reproducible, labour-intensive, and time-consuming (typically taking more than 7 days to complete) [8]. Although pseudovirus-based neutralization tests (pVNT) have been developed to mitigate biosafety risks, they still rely on specialized cell culture and relatively long incubation periods (48–72 h). Furthermore, both cVNT and pVNT are limited in throughput, making them unsuitable for rapid, large-scale epidemiological studies, population screening and the real-time monitoring of immune recovery in clinical settings.

**Figure 1.**
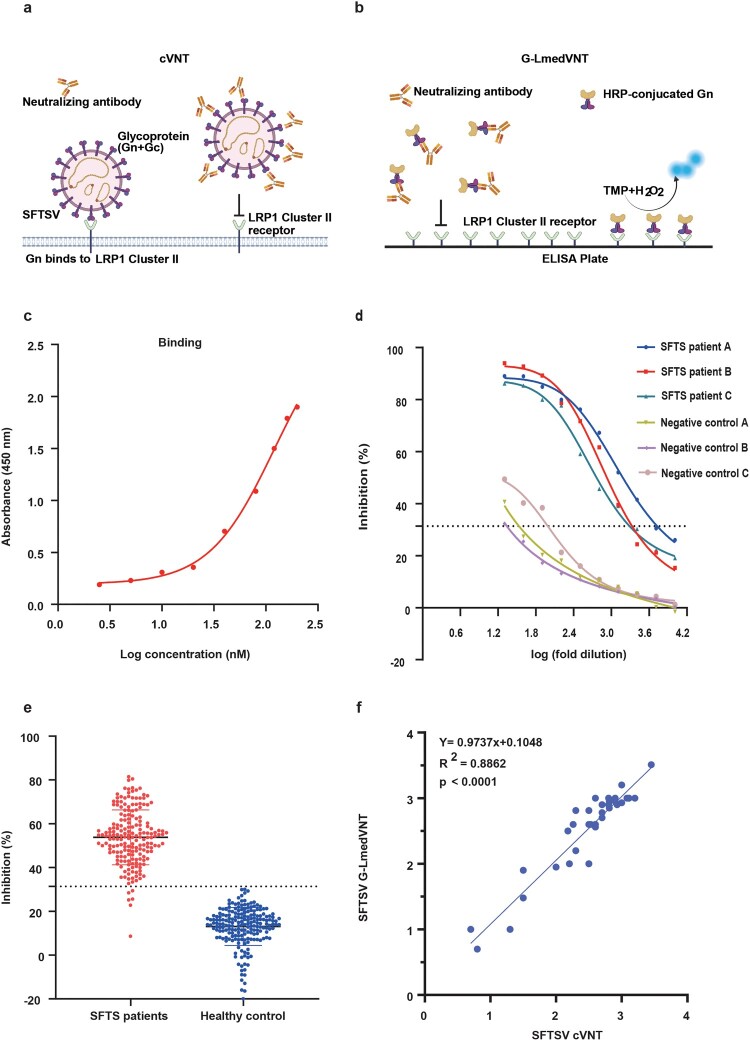
Gn–LRP1 mediated virus neutralization test against SFTSV. (a) The mechanism of cVNT. Anti-SFTSV neutralizing antibodies block the SFTSV Gn from binding to LRP1 receptor proteins on the host cell surface. (b) In the Gn–LRP1 mediated virus neutralization test (G-LmedVNT), anti-SFTSV neutralizing antibodies block HRP-conjugated Gn protein from binding to the LRP1 protein pre-coated on an ELISA plate. The illustrations were created using BioRender. (c) Binding of HRP-conjugated SFTS-Gn proteins to LRP1. (d) Inhibition of SFTS-Gn-LRP1 interaction by plasma from patients with SFTS. (e) Neutralizing antibodies testing of SFTS patient cohort (*n* = 190) and healthy cohorts (*n* = 200) at a 1:320 dilution. (f) Correlation analysis for 50 SFTS plasma samples with different levels of SFTS nAbs by G-LmedVNT and cVNT.

Recent studies have identified LRP1 as the primary functional receptor for SFTSV entry [9], proving an important foundation for this work. Our team previously pioneered the rapid development of surrogate virus neutralization test for SARS-CoV-2 based on antibody-mediated block of virus-receptor interaction, mimicking the live virus entry of cells [10]. The cPass kit for SARS-CoV-2 became the first FDA-approved VNT for any viruses and it has been deployed in more than 90 countries. Using the same principle, here we describe the rapid development of the Gn–LRP1 mediated virus neutralization test (G-LmedVNT) for SFTSV, analogous to the mechanism utilized by cVNT with live virus (Figure 1a,b). This G-LmedVNT overcomes the technical bottlenecks of cVNT by eliminating the need for live viruses or cells, enabling results within 1–2 h in comparison to several days required for cVNT and pVNT.

## The study

The test uses purified SFTSV receptor-binding glycoprotein N (Gn) and the host cell receptor LRP1, mimicking virus-host interactions within an ELISA system. This Gn-LRP1 interaction can be blocked or neutralized by specific nAbs in patient plasma, in the same manner as in cVNT. In this study, the SFTSV Gn (GenBank accession no. MT522609) and LRP1 Cluster II genes were commercially synthesized by GenScript (Nanjing, China) and cloned into the pcDNA3.4 expression vectors, recombinant proteins were expressed using the Chinese Hamster Ovary (CHO-S) suspension cell expression system (refer to Supplementary Material for details). The sequence information for these recombinant proteins was shown in Supplementary Table 1. The purified proteins were characterized by SDS-PAGE and SEC-HPLC to assess its purity and molecular weight. Protein concentration was determined using a NanoDrop 2000 spectrophotometer (Thermo Fisher Scientific, USA). The Gn protein was conjugated to horseradish peroxidase (HRP) using the Lightning-Link® HRP Conjugation Kit (ab102890, Abcam, Cambridge, UK) according to the manufacturer’s instructions.

For direct binding assay, an ELISA plate (Labgic Technology Co., Ltd., Hefei, China) was pre-coated with LRP1 Cluster II protein (GenScript) at 400 ng per well in 50 μL of 100 mM carbonate–bicarbonate coating buffer (pH 9.6) overnight at 4°C, followed by blocking with 2% bovine serum albumin (BSA) (MedChem Express, USA). HRP-conjugated Gn was added to the LRP1-coated plate at different concentrations in 100 μL of PBST (PBS containing 0.05% Tween-20) containing 2% BSA for 1 h at room temperature. Unbound antigens were removed by five washes with phosphate-buffered saline, PBST. A colorimetric signal was developed on the enzymatic reaction of HRP with a chromogenic substrate, 3,3”,5,5'-tetramethylbenzidine (TMB) (Elabscience Bionovation Inc., Wuhan, China). An equal volume of TMB stop solution was added to stop the reaction, and the absorbance readings at 450 nm were acquired using a Spark multimode microplate reader (Tecan, Switzerland).

For the validation of the G-LmedVNT assay, we used a panel of 390 human plasma samples, comprising 200 samples from healthy controls and 190 samples from laboratory-confirmed SFTS patients (≥7 days post-symptom onset), all of whom survived (Supplementary Material). All plasma samples were heat-inactivated at 56°C for 30 min before testing to eliminate complement interference. HRP-Gn (600 ng) was pre-incubated with test plasma for 1 h at 37°C in a final volume of 50 μL, followed by addition into the ELISA plate pre-coated with LRP1 Cluster II protein (500 ng per well, as described above) for 1 h at room temperature. Unbound HRP-conjugated antigens were removed by PBST washes. Level of neutralization is expressed as inhibition (%) = (1-sample optical density value/negative control optical density value) × 100. For determination of neutralization titre, human plasma was used with a twofold serial dilution starting at 1:20, the same as for cVNT described below. Same was done for the control samples.

As shown in Figure 1c, there is a clear dose-dependent and specific binding between LRPl and Gn. We then demonstrated that this specific Gn-LRPl binding can be blocked or neutralized by SFTS patient plasma in a dose-dependent manner (Figure 1d). To determine the optimal serum dilution for the G-LmedVNT in SFTS patients, we evaluated a range of serial dilutions (1:20–1:320) to detect the nAbs of SFTS patients, and healthy control. While all tested dilutions demonstrated excellent discriminative power with Area Under the Curve (AUC) values exceeding 0.99 (Supplementary Fig), a 1:320 dilution was identified as optimal, as it effectively eliminated the matrix effect and non-specific interference observed at lower dilutions and also provided the highest AUC (Figure 1e, Supplementary Fig). At this dilution, the overall AUC reached 0.995 (95% CI: 0.986–1.000). The optimal cut-off value was set at 31.35%, resulting in a sensitivity of 96.79% and a specificity of 100%. By optimizing the plasma dilution to achieve a maximum signal-to-noise ratio, we ensure that the observed inhibition accurately reflects specific neutralizing activity rather than non-specific binding events.

To ensure the technical robustness of the G-LmedVNT platform for high-throughput screening, we evaluated its intra-assay and inter-assay precision. As summarized in Supplementary Table 2, the intra-assay coefficients of variation (CVs) ranged from 0.95% to 9.28%, while the inter-assay CVs ranged from 0.35% to 9.60%. These results demonstrate that variability in the G-LmedVNT assay remains well within acceptable limits for diagnostic use, supporting its reliability and reproducibility.

The performance of the G-LmedVNT was further evaluated through a head-to-head correlation analysis with the cVNT. The cVNT (50% plaque reduction neutralization test) assay was conducted as previously described [8, 11]. A sub-panel of 50 SFTS plasma samples spanning a broad range of nAb levels was selected, including low titres (IC50 < 500, *n* = 18), medium titres (500 ≤ IC50 < 1000, *n* = 12), and high titres (IC50 > 1000, *n* = 20) (Supplementary Table 3). As shown in Figure 1f, the two assays demonstrated good overall correlation. Linear regression analysis yielded an *R^2^* of 0.8902, indicating that the G-LmedVNT as a reliable surrogate for measuring nAbs against SFTSV.

## Discussion

Serological nAb detection is critical for assessing individual immune status and comprehensive public health applications, including evaluating the effectiveness of a vaccine, determining immunity after natural infection, assessing asymptomatic infection rates, and identifying natural and intermediate hosts of the virus for risk assessment and source control [12, 13]. The G-LmedVNT platform presented in this study offers a safe, rapid, and high-throughput alternative to cVNT and pVNT, enabling precise quantification of nAbs in SFTS patients. This provides a practical tool for assessing individual immune status and monitoring recovery in large-scale clinical screening. In clinical settings or family clusters where non-vector-borne transmission is suspected, the G-LmedVNT can be deployed to screen large cohorts of close contacts. By identifying subclinical or asymptomatic cases that may be missed by RT–PCR, this high-throughput assay facilitates more accurate reconstruction of human-to-human transmission chains and improves understanding of viral virulence. Furthermore, as SFTSV is classified as a BSL-3 pathogen outside of China, the G-LmedVNT platform significantly lowers the barrier for global surveillance.

Building upon the established framework for SARS-CoV-2 [10], this assay detects total nAbs targeting the critical receptor-binding interface without relying on species-specific secondary antibodies, making it inherently species-independent. This biochemical advantage, as demonstrated in diverse animal models by Tan et al. [10], makes the G-LmedVNT particularly well suited for large-scale ecological surveillance across wildlife and livestock. As such, it can serve as a critical tool for elucidating SFTSV maintenance mechanisms and mitigating spillover risk by enabling seroprevalence mapping across multiple species.

Several limitations of this study should be acknowledged. First, experimental validation in animal reservoirs or non-human primate models was not performed due to limited access to BSL-3 animal facilities and challenges in obtaining confirmed positive animal sera during the study period. Second, although LRP1 has been identified as a key functional receptor for SFTSV entry, the potential involvement of unidentified co-receptors may limit the G-LmedVNT’s ability to detect the complete spectrum of nAbs. Lastly, the G-LmedVNT may not detect nAbs that target epitopes outside the LRP1-Gn interaction interface.

## Supplementary Material

Supplementary Material.docx

## Data Availability

The datasets used during the current study are publicly available and can also be obtained from corresponding authors on request.
