# Supplementary material for: A novel assay for quantifying Gn–LRP1–mediated neutralizing antibodies against severe fever with thrombocytopenia syndrome virus
Source: Emerg Microbes Infect. 2026 Apr 24;15(1):2665001. doi: 10.1080/22221751.2026.2665001 (PMC13435290; doi:10.1080/22221751.2026.2665001)
Supplement: Supplementary file 1 [file TEMI_A_2665001_SM9476.docx]

#### Supplementary Material

#### Methods

#### 1. Expression, and Purification of SFTSV Gn and LRP1 Cluster II **proteins**

#### **1.1** Expression Plasmid Construction

The SFTSV Gn and LRP1 Cluster II genes were cloned into the pcDNA3.4 expression vectors.

#### **1.2** Protein Expression in CHO-S Cells

Recombinant Gn protein was produced in suspension-adapted CHO-S cells. Cells were cultured in serum-free medium in Erlenmeyer flasks at 36.5°C in a humidified incubator with 5% CO₂ while shaking at 120 rpm. One day before transfection, cells were seeded at a density of 1.0 × 10⁶ cells/mL. Transient transfection was carried out using [polyethylenimine (PEI) / a commercial transfection reagent] according to the manufacturer's protocol. Enhancers and feeds were added at specific time points post-transfection to prolong culture viability and increase yield. The culture supernatant was harvested by centrifugation (4,000 × g for 20 min at 4°C) when cell viability fell below 80%. The same as LRP1 Cluster II protein.

#### **1.3** Purification of Recombinant Gn

To obtain highly pure monomeric protein, a two-step chromatography strategy was employed. First, for affinity capture, the clarified supernatant was incubated with Ni-NTA Magnetic Agarose Beads (GenScript) for 4 h at room temperature with gentle rotation. The beads were separated using a magnetic stand and washed three times with washing buffer (25 mM Tris-HCl, 300 mM NaCl, pH 8.0), and bound protein was eluted with elution buffer (25 mM Tris-HCl, 300 mM NaCl, 500 mM Imidazole, pH 8.0). Subsequently, preparative size-exclusion chromatography (SEC) was performed by loading the concentrated eluate onto a Chromdex 200 PG (320 mL) prepacked column (Bestchrom, Shanghai, China) pre-equilibrated with PBS (pH 7.2). Chromatography was conducted at a flow rate of 3.9 mL/min using an ÄKTA pure system (Cytiva), and elution was monitored at 280 nm. Fractions corresponding to the monomeric peak were collected, pooled, and concentrated to the desired final concentration. The purified monomeric protein was aliquoted and stored at –80°C until further analysis.

#### **1.4** Quality Control Analysis

The purified protein was analyzed by **SDS-PAGE** under reducing and non-reducing conditions, followed by Coomassie Blue staining. The homogeneity and aggregation state were analyzed by **SEC-HPLC** using a [TSKgel G3000SWXL column (7.8 mm × 30 cm, 5 µm, Tosoh Bioscience, Tokyo, Japan)] column. Protein concentration was determined using a NanoDrop spectrophotometer based on the theoretical extinction coefficient.

2. **Inclusion Criteria for SFTS Patients and Exclusion Criteria**

**2.1 Inclusion Criteria for SFTS Patients:**

**2.1.1** SFTS was confirmed based on at least one of the following criteria: (1) positive SFTSV nucleic acid test; (2) concurrent positivity for SFTSV IgM and IgG; or (3) a ≥ 4-fold rise in IgG antibody titers between acute and convalescent phases. All of the SFTS patients survived at last.

**2.1.2** Sample collection timing ≥ 7 days after the onset of symptoms to ensure detectable antibody levels were included.

**2.2 Exclusion Criteria:** Other potential febrile illnesses with similar clinical manifestation, such as human granulocytic anaplasmosis, scrub typhus, or other viral hemorrhagic fevers, were excluded.
